# Fast dynamic ventilation MRI of hyperpolarized ^129^Xe using spiral imaging

**DOI:** 10.1002/mrm.26912

**Published:** 2017-09-16

**Authors:** Ozkan Doganay, Tahreema N. Matin, Anthony Mcintyre, Brian Burns, Rolf F. Schulte, Fergus V. Gleeson, Daniel Bulte

**Affiliations:** ^1^ Department of Oncology University of Oxford, Old Road Campus Research Building, Roosevelt Drive Oxford OX3 7DQ United Kingdom; ^2^ Department of Radiology, The Churchill Hospital Oxford University Hospitals NHS Trust, Old Road Headington OX3 7LE United Kingdom; ^3^ GE Global Research Munich Germany

**Keywords:** hyperpolarized ^129^Xe, dynamic MRI, lung, k‐space sampling, spiral, signal‐time curves, COPD

## Abstract

**Purpose:**

To develop and optimize a rapid dynamic hyperpolarized ^129^Xe ventilation (DXeV) MRI protocol and investigate the feasibility of capturing pulmonary signal‐time curves in human lungs.

**Theory and Methods:**

Spiral k‐space trajectories were designed with the number of interleaves *N*_int_ = 1, 2, 4, and 8 corresponding to voxel sizes of 8 mm, 5 mm, 4 mm, and 2.5 mm, respectively, for field of view = 15 cm. DXeV images were acquired from a gas‐flow phantom to investigate the ability of *N*_int_ = 1, 2, 4, and 8 to capture signal‐time curves. A finite element model was constructed to investigate gas‐flow dynamics corroborating the experimental signal‐time curves. DXeV images were also carried out in six subjects (three healthy and three chronic obstructive pulmonary disease subjects).

**Results:**

DXeV images and numerical modelling of signal‐time curves permitted the quantification of temporal and spatial resolutions for different numbers of spiral interleaves. The two‐interleaved spiral (*N*_int_ = 2) was found to be the most time‐efficient to obtain DXeV images and signal‐time curves of whole lungs with a temporal resolution of 624 ms for 13 slices. Signal‐time curves were well matched in three healthy volunteers. The Spearman's correlations of chronic obstructive pulmonary disease subjects were statistically different from three healthy subjects (*P* < 0.05).

**Conclusion:**

The *N*_int_ = 2 spiral demonstrates the successful acquisition of DXeV images and signal‐time curves in healthy subjects and chronic obstructive pulmonary disease patients. Magn Reson Med 79:2597–2606, 2018. © 2017 The Authors Magnetic Resonance in Medicine published by Wiley Periodicals, Inc. on behalf of International Society for Magnetic Resonance in Medicine. This is an open access article under the terms of the Creative Commons Attribution License, which permits use, distribution and reproduction in any medium, provided the original work is properly cited.

## INTRODUCTION

Functional pulmonary imaging techniques including hyperpolarized (HP) gas (^3^He and ^129^Xe) MRI are a growing field for the non‐invasive assessment of regional lung function 1, 2, 3, 4. Hyperpolarized gas MRI enables the evaluation of ventilation and gas diffusion, which is typically carried out in a static imaging fashion during a breath‐hold interval, following inhalation of ^3^He or ^129^Xe, and demonstrates homogeneous signal intensity in healthy lung regions 5, 6, 7, 8. Regions of absent or relatively low signal are known as “ventilation defects” and correspond to regions of obstructed airflow 9, 10. Dynamic ventilation imaging with HP gas MRI is possible during a complete breath cycle, including the inhalation and exhalation intervals, using fast non‐Cartesian k‐space sampling strategies 11, 12, 13, 14, 15. Previously, HP ^3^He dynamic MRI with an interleaved‐spiral readout was reported to be sensitive to regional signal‐time curves in six healthy volunteers and six patients with lung disease, including severe asthma, emphysema, and cystic fibrosis 16. With wider availability and lower cost than ^3^He, regional lung function assessment with HP ^129^Xe‐MRI offers a more clinically viable technique. In addition, ^129^Xe chemical shift‐based spectroscopy and imaging approaches provide a unique opportunity to derive regional functional information about gas transfer and exchange dynamics because of the inherent solubility and chemical shift of ^129^Xe 17, 18, 19. The purpose of this study was to develop and optimize a multi‐slice dynamic HP ^129^Xe ventilation (DXeV) imaging technique and investigate the feasibility of capturing signal‐time curves using an interleaved‐spiral k‐space sampling strategy during a complete breath cycle.

To capture dynamic gas flow using HP ^3^He ventilation imaging, a temporal resolution of hundreds of milliseconds is required. Although this has been successfully achieved using echo‐planar imaging‐based methods, the images contained significant blooming artefacts 20. Blooming artefacts have been reduced by using an interleaved spiral k‐space sampling strategy 16, 21, which has the additional advantage of improved temporal resolution over standard Cartesian ^3^He ventilation imaging 22. Susceptibility artefacts on dynamic ^3^He ventilation images were reported to be less apparent with a higher number of interleaves 16, 23. A radial acquisition strategy has previously been reported to be superior for susceptibility artifact reduction when a larger number of views (i.e., more interleaves) is used 11. However, the larger number of views limited both the SNR and the temporal resolution when capturing the dynamics of gas ventilation, because of the use of lower flip angles and gas‐inflow effects 24, 25, 26.

The relatively long transverse relaxation time ( $T_{2}^{*}$) of ^129^Xe in the gas phase ( $T_{2}^{*}$ = 52 ± 20 ms at 1.5T) 27 compared to ^3^He ( $T_{2}^{*}$ = 27.8 ± 1.2 ms at 1.5T) 28 allows the use of longer readout times per interleave, fewer radiofrequency (RF) pulses, and larger flip angles 18. The use of fewer interleaves allows for rapid coverage of the whole of k‐space, resulting in higher temporal resolution in dynamic gas ventilation imaging. In this study, we describe the successful application of a multi‐slice DXeV imaging technique in human lungs. The feasibility of capturing signal‐time curves using DXeV imaging during inhalation, short breath‐hold (∼5s), and exhalation intervals was determined in healthy and chronic obstructive pulmonary disease (COPD) subjects. The temporal and spatial resolution of signal‐time curves were also investigated in a gas‐flow phantom and compared to finite element modelling of gas‐flow.

## THEORY

### Dynamics of HP ^129^Xe MR Signal

The loss of HP ^129^Xe gas signal as a function of a train of RF pulses is modelled as a static system where the total concentration of HP ^129^Xe is constant within a breath‐hold interval 29. Therefore, signal loss from a gas phantom filled with a certain amount of HP gas is proportional to the number of RF pulses, the flip angle, and the longitudinal relaxation time, T_1_ 30, 31. However, in the case of dynamic ventilation imaging, the HP gas signal also depends on the concentration of ^129^Xe gas as a function of time because of the gas‐inflow effects, which incorporate ^129^Xe gas convection and diffusion 26, 32, 33. Therefore, the equation governing the loss of HP ^129^Xe gas signal for dynamic ventilation gas imaging is given by:

(1) $$S=A\times T\left(\alpha \left(r\right)\right)\times [\cos^{n-1}\left(\alpha \right)\times \sin\left(\alpha \right)]\times \exp\left(-\frac{t}{T_{1}}\right)\times \exp\left(-\frac{\text{TE}}{T_{2}^{*}}\right)\times C_{xe}(r,t).$$

Where *A* is a scaling constant that is dependent on ^129^Xe polarization levels, coil sensitivity, receive gain, and the point spread function, which depends on the k‐space sampling strategy (i.e., spiral, radial, Cartesian) 18, 34. *T* is transmit profile of the RF coil where the flip angle, α, changes as a function of the position, *r* 35. The term $\left[\cos^{n-1}\left(\alpha \right)\times \sin\left(\alpha \right)\right]$ is associated to the RF depolarization because of the excitation of magnetization as a function of the flip angle, α and the number of RF pulses,  n 18, 23. The first exponential term is longitudinal relaxation where  t is the imaging scan time when ^129^Xe gas is within the lungs and T_1_ is the time constant for the HP magnetization 36 within the lungs to relax to thermal equilibrium. The second exponential term is the transverse relaxation where $\;T_{2}^{*}$ is the transverse relaxation decay time constant and TE is the echo time. $C_{Xe}$ is the volume concentration of ^129^Xe gas within a region of interest (ROI) related to bulk‐flow (i.e., convection) and ^129^Xe gas diffusion as a function of time because of the different ^129^Xe gas arrival times to different ROIs. The dynamic concentration of ^129^Xe gas, $\;c_{Xe}\left(r,t\right)$ must be calculated spatially and temporally depending on the gas‐flow rate.

## METHODS

All images were acquired using a 1.5T MRI system (Signa HDx, GEHC, Milwaukee, WI) and a flexible vest‐shaped transmit‐receive RF coil (Clinical MR Solutions, Brookfield, WI). A commercial polarizer system (Model 9300, Polarean, Durham, NC) was used to polarize 86% nuclear enriched ^129^Xe gas using a flow rate of 1 L/h. ^129^Xe polarization levels of 10–15% were achieved. Polarization was measured using a commercial polarization measurement station (Model 2881, Polarean, Durham, NC).

### Design of the k‐Space Trajectory

Spiral trajectories were designed to achieve an optimal spatial resolution and signal‐to‐noise ratio (SNR) for each interleave strategy within the limits of clinical slew rates of 200 T/m/s and a gradient amplitude of 50 mT/m. The T_read_ per interleaf and the pixel resolutions are shown in Table 1 for 1‐interleaf, 2‐interleaf, 4‐interleaf, and 8‐interleaf spirals (i.e., *N*_int_ = 1, 2, 4, 8). This choice of spiral interleaved strategy resulted in comparable $T_{2}^{*}$ blurring artefacts (<∼10% of the available spatial resolution) per interleaf. DXeV images were reconstructed with regridding and density weighting with a line broadening of 5Hz as described in Wiesinger et al. 37 and Schulte et al. 38.

### Design of Gas‐Flow Phantom

**Figure 1:**
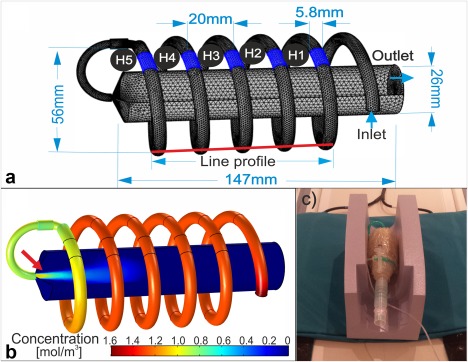
(**a**) Simulation geometry of the gas‐flow phantom, including the line profile along the helix tube (red solid line), the syringe body, ROIs from H1 to H5, inlet and outlet boundaries, and tetrahedral mesh (minimum and maximum element sizes are 0.2 mm and 2.3 mm). (**b**) Corresponding concentration map of ^129^Xe, *c_Xe_* (*r,t*), at a time point of 3.5 s post activation. (**c**) The gas‐flow phantom. Principle dimensions of the phantom are shown in (**a**). Arrow in (**b**) shows gas bulk flow effect.

A gas‐flow phantom was built to investigate the temporal and spatial resolution of DXeV imaging. Simulation geometry and a picture of the phantom are shown in Figure 1. A gas sampling tube with an internal diameter of 5.8 mm was wrapped around a syringe tube (60 mL) in the form of a helix as shown in Figure 1a. The diameter of the syringe body was 26 mm. The syringe body was wrapped with a 15‐mm thick plastic cover to increase the major diameter of the helix tube to 56 mm. The helix tube diameter and the pitch length may vary ±2 mm compared to the simulated phantom geometry because the tubing and the plastic cover were flexible materials as shown in Figure 1c. ^129^Xe gas in the reservoir tube was delivered to the syringe through an extension gas sampling tube (M11145070, Helsinki, Finland, GE Healthcare) using an MRI contrast agent power injector (92901‐T‐153 Rev, MedRad, Warrendale, PA). The maximum reservoir gas‐volume and gas‐flow rate of the power injector were 60 ml and 10 ml/s, respectively. This allowed a gas flow rate within the helix tube at ROIs from H1 to H5 of ∼250 ml/s: (1) similar to the gas‐flow rate used in the FEM simulation model, and (2) comparable to the gas‐flow within the trachea when a liter of gas is inhaled within 4s 39.

### Gas‐Flow Phantom Imaging

**Figure 2:**
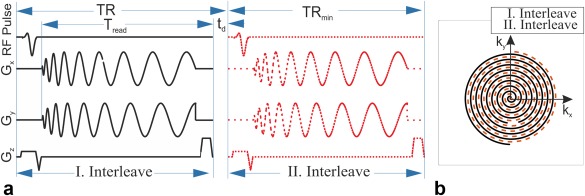
Pulse sequence timing diagram for *N*_int_ = 2 spiral (**a**) I. Interleave is sampled following an RF pulse. (**b**) II. Interleave plays after a time delay (*t*_d_). I and II interleaves cover different locations of k‐space as shown in (**b**). *G*_x_ and *G*_y_ are x and y gradients. T_read_ is the k‐space readout duration. TR_min_ is the time duration for a single interleave including the time required for the RF pulse, slice select gradient, *G*_x_, *G*_y_, and the spoiler. TR is pulse repetition time including *t*_d_ that is also a time delay between the first interleave and second interleave given by number of slices times TR_min_.

Single‐slice DXeV images of the gas‐flow phantom were carried out in a coronal plane with a slice thickness of 10 mm that was selected at the center of the phantom including ROIs from H1 to H5 (as shown in Fig. 1a). The pulse sequence timing diagram is shown in Figure 2a for *N*_int_ = 2. Twelve sets of DXeV images were acquired with pulse repetition time (TR) = 0.5 s for *N*_int_ = 1 and *N*_int_ = 2 resulting in temporal resolution of 0.5 s and 1 s for each scan, respectively. Although shorter TR values for single‐slice phantom imaging were possible, the temporal resolutions were selected by corresponding to the TR values approximately achievable with in vivo multi‐slice imaging. For each scan, the power injector was filled with 60 ml of HP ^129^Xe gas and started simultaneously with the MR scanner (±0.5 s). To investigate the spatial resolution, DXeV images were obtained with *N*_int_ = 1, 2, 4, and 8, for a field of view of 15 cm corresponding to voxel sizes of 8 mm, 5 mm, 4 mm, and 2.5 mm, respectively. The total number of RF pulses used was *N* = 12, 24, 48, and 96 for *N*_int_ = 1, 2, 4, and 8 to achieve 12 sets of single‐slice DXeV image, respectively. Therefore, the optimal flip angle, *α*_*opt*_, was calculated using a constant flip angle approach for the static system that maximized the SNR for the very last image as previously proposed by Miller et al. 30 as shown in Table 1. Additionally, for *N*_int_ = 2, the interleaved I and II trajectories as shown in Figure 2b were reconstructed as two separate images to investigate increased acquisition efficiency similar to the concept of a spiral‐in/‐out *k*‐space reconstruction 40.

**Table 1:** Summary of Parameters Used to Design Interleaved Spiral Sequences and Images

*N*_int_	Pixel Resolution (mm)	TR_min_ (ms)	T_read_ (ms)	α (°)	Temporal Resolution (ms)
1	16	28	18	19	390
2	10	24	14	12	624
4	8	23	13	8	1196
8	5	23	13	6	2392

*N*_int_ is the number of interleaves for sampling k‐space. The pixel resolution excluding the $T_{2}^{*}$ blurring was reported for FOV = 30 cm. TR_min_ includes a default selection of 5 ms time duration for the spoiling gradient and 4 ms slice selection to the read‐out time. T_read_ is the readout time for sampling the k‐space data per interleaves. α is the flip angle. The temporal resolution is calculated by *N*_int_ × TR_min_ × *N*_sl_. *N*_*sl*_ is the number of slices.

**Table 2:** Summary of COPD Study Population

Subject No.	Sex (M/F)	Age (y)	GOLD stage	FEV_1_ (% predicted)	FEV_1_/FVC (% predicted)
1 (OXF0086)	M	72	II	71	67
2 (OXF0055)	M	74	III	32	43
3 (OXF0144)	M	69	III	41	53

F, female; M, male; FEV_1_, forced expiratory volume in 1 second; FVC, forced vital capacity.

### Experimental Signal‐Time Curves

Experimental signal‐time curves from H1 to H5 were calculated by taking the mean signal intensity of ROIs on DXeV images (as shown in online supporting files; see Supporting Fig. S1) and then compared to the corresponding simulated signal‐time curves. Line profiles from H1 to H5 (Fig. 1a) were calculated from the images taken at a time of *t* = 6 s to allow direct comparison of the spatial‐resolution between *N*_int_ = 1, 2, 4, and 8. The full width at half maximum (FWHM) of the line profiles were correlated to the spatial resolution.

### Numerical Modelling of $c_{Xe}\left(r,t\right)$

A simulation geometry was built with similar dimensions to the gas‐flow phantom geometry as shown in Figure 1a. This enabled quantitative comparison of signal‐time curves between simulated and real images. The simulation geometry consisted of three components including an extension tube, a helix tube along the line profile (Fig. 1a), and the syringe body as described in the Methods section. ^129^Xe gas was administered through the extension tube and helix tube, within the imaging field of view along the line profile and syringe body as shown in Figure 1a. $c_{Xe}\left(r,t\right)$ was simulated for the gas‐flow geometry in two steps as follows: 1 the velocity field of ^129^Xe, $\;u_{Xe}$, was calculated from the solution of the Navier‐Stokes equations using the laminar flow regime in the steady state, and 2 $c_{Xe}\left(r,t\right)$ was calculated by means of diffusion and convection equations as a function of time including $u_{Xe}$ from the first step 41. In step 1, a gas‐flow rate of 250 ml/s within the helix tube was selected corresponding to the flow‐rate of gas in the trachea when a liter of gas is inhaled over 4 s. This was achieved by selecting a velocity of ∼0.4 m/s at the inlet boundary of the extension pipe as shown in Figure 1a. Gas flows through the extension tube, then through the helix tube, enters the syringe body, and leaves the geometry through the outlet channel. In step 2, the inward flux at the inlet boundary was selected to be 0.45 mol/m^2^s corresponding to the phantom DXeV images. The laminar flow regime was chosen by calculating the Reynolds number to be 599 for the inlet boundary; considering the inlet velocity to be 0.4 m/s, the density of xenon gas is 5.89 kg/m^3^ and dynamic viscosity is 2.28 × 10^−5^ Ns/m^2^ 42. The driving force for the xenon gas was diffusion by Fick's law for a diffusion coefficient of 0.14 × 10^−4^ m^2^/s 43 and the convection knowing the $u_{Xe}$. The solution of step 1 from the Laminar Flow Physics Interface was coupled to the solution step 2 Transport of Diluted Species interface in COMSOL Multiphysics software (Version 5.2, Burlington, MA) and yielded the 3D distribution of $c_{Xe}\left(r,t\right)$.

### Simulated Signal‐Time Curves

Simulated $c_{Xe}\left(r,t\right)$ was calculated for a step size of 0.1 s to represent the ideal case and 0.5 s for comparison to the TR that was used in the phantom imaging over a time period of 6 s related to the experimental protocol as explained in the Methods section. To calculate $\;C_{Xe}$, simulated $\;c_{Xe}\left(r,t\right)$ was integrated for each ROI, H1 to H5 (as shown in Fig. 1a), with the volume integral limits set to the slice thickness (10 mm) of the RF pulse used in DXeV imaging of the gas‐flow phantom. By substituting $C_{Xe}$ into Eq. (1), simulated signal‐time curves were then derived from H1 to H5 for *N*_int_ = 1, which includes the effects of the number of RF pulses (N = 1:12), flip angle (α = 19°), T_1_ = 200 s 44, and $T_{2}^{*}$ = 25 ms 45 in the phantom. The flip angles, $T_{2}^{*}$, and T_1_ between ROIs were assumed to be constant in Eq. (1). For quantitative analysis of the temporal resolution and gas‐inflow effects, simulated signal‐time curves were compared to the experimental signal‐time curves for *N*_int_ = 1. Nonetheless $T_{2}^{*}$ was not measured and assumed to be 25 ms for the phantom imaging, the effect of exp(−TE/ $T_{2}^{*}$) term was negligible because $T_{2}^{*}$ > > TE = 50 µs.

### In Vivo Imaging

Multi‐slice DXeV images were acquired with an *N*_int_ = 2 spiral from six subjects, three healthy (male, 43, 34, and 31 years of age) and three COPD subjects to investigate the feasibility of capturing signal‐time curves. Patients with COPD were recruited as part of a study approved by the NRES Committee South Central (Berkshire, REC reference 11/SC/0488), and written informed patient consent was obtained. Summary of COPD study population is shown in Table 2. Subjects were instructed to inhale 1 L of HP ^129^Xe gas from a Tedlar plastic bag (Jensen Inert Products, Coral Springs, FL) within ∼3 s during an inhalation period, followed by a 5‐s breath‐hold, and then exhale the gas over 3 s during the exhalation period. DXeV images were acquired over a total scan time of 20 s, including a baseline‐period of ∼2 s before the inhalation‐period and a flush‐interval of 5 s after the exhalation‐period. A Gaussian‐shaped slice selective RF pulse was used to achieve a 15‐mm slice thickness, which allowed for full coverage of the lungs using 13 coronal slices. DXeV images were obtained using a conventional multi‐slice imaging scheme in which all slices within the imaging volume were sequentially collected for each interleaf. For example, considering TR_min_ was 24 ms as shown in Figure 2a, the time required to obtain 13 slices was 312 ms for the I interleaf, resulting in a total scan time of 624 ms at the end of the II interleaf spiral. This resulted in a TR of 312 ms and a time delay, t_d_ = TR‐TR_min_, of 288 ms between I and II interleaves as shown in Figure 2a for the conventional multi‐slice imaging. This timing approach allowed 32 DXeV image volume sets to be acquired during a total scan time of ∼20 s. Alpha (α) was selected to be 10° for in vivo imaging to maximize the signal for the very last image at the end of the breath‐hold as explained in the phantom imaging subsection.

### In Vivo Signal‐Time Curves and Statistical Analysis

Signal‐time curves were then obtained from ROIs selected in the mid zones of the left and right lung and in the trachea. The signal‐time curves were compared between a selected ROI (Slice 2‐left) and other ROIs for each subject. The signal‐time curves at ROIs from left and right were expected to be influenced by the subjects' breathing because each subject may breath slightly differently than each other. Therefore, the ROI trachea signal is considered as an input boundary condition representing the effects of the subjects' breathing for comparision across the subjects. The Spearman partial correlation analysis was used to include signal‐time curves from trachea to eliminate the difference between patient‐breathing efforts. The signal‐time curve from ROI (Slice 2‐left), other ROIs, and ROI trachea were described as the first, second, and controlling variables, respectively. Therefore, the Spearman partial correlation rho values were calculated relative to Slice 2‐left for each subject while controlling for ROI trachea.

A paired samples *t* test was used to investigate the statistical difference between rho values across the heathy subjects to investigate whether the signal‐time curves are repeatable for the healthy lungs. The mean of healthy volunteer rho values was then compared to COPD subjects to investigate whether the difference between heathy and COPD subjects was statistically significant within 95% confidence interval. The flip angles were calculated in healthy subjects by fitting the term of RF depolarization in Eq. (1) to the breath‐hold data similar to Hahn et al. 15 assuming $T_{2}^{*}$ = 25 ms, T_1_ = 20 s 46, $c_{Xe}\left(r,t\right)$ are spatially constant (i.e., static regime).

## RESULTS

The geometry of the gas‐flow phantom that was used to obtain simulated $c_{Xe}\left(r,t\right)$ and DXeV phantom images are shown in Figure 1a. A simulated $c_{Xe}\left(r,t\right)$ map is shown in Figure 1b for the 3.5‐s time point after the power injector was activated. ^129^Xe gas is shown to travel along the line profile within the helical tube with decreasing concentration from H1 to H5. As can be seen, $\;c_{Xe}\left(r,t\right)\;$ within H1, H2, and H3 have nearly reached the concentration of xenon gas at the inlet boundary and is beginning to enter into the syringe body. The gas‐flow along the central core of the syringe body demonstrates bulk flow into the syringe body resulting in a relatively high $c_{Xe}\left(r,t\right)\;$ at the center (Fig. 1b).

**Figure 3:**
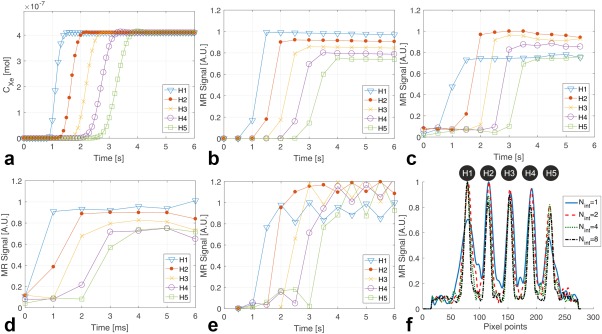
(**a**) Simulated ***C*_*Xe*_** from H1 to H5 with time steps of 0.1 s over a time point of 6 s after post activation. (**b**) Simulated signal time curves for *N*_int_ = 1 spiral with time steps of 0.5 s. (**c**) Experimental signal time curves from *N*_int_ = 1 spiral and TR = 0.5 s. (**d**) Experimental signal time curves from *N*_int_ = 2 spiral TR = 0.5 s. (**e**) Experimental signal time curves from *N*_int_ = 2 spiral after reconstruction of interleaves I and II as two separate images. (**f**) Line profiles of DXeV images obtained from H1 to H5 for *N*_int_ = 1, 2, 4, and 8.

Simulated values of $C_{Xe}$ from H1 to H5 are shown in Figure 3a with a time step size of 0.1 s over a total simulation time of 6 s. The concentrations of ^129^Xe gas at H2 and H5 saturate at ∼2 s and 4 s, respectively. The gas arrival time from one ROI to the next ROI takes ∼0.5 s. Therefore, simulated signal‐time curves from H1 to H5 are derived for the *N*_int_ = 1 spiral from Eq. (1) for *N* = 1 to *N* = 5, respectively, as shown in Figure 3b with a time step of 0.5 s over a total simulation time of 6 s similar to the DXeV images of phantom. Although the $c_{Xe}\left(r,t\right)$ at H1 and H5 is the same after 4 s, the simulated signal‐time curve amplitude at H5 is lower than H1 because of the HP gas at H5 having experienced five RF excitations during the gas transit time. Experimental signal‐time curves from H1 to H5 using the *N*_int_ = 1 spiral are also shown in Figure 3c. As expected, experimental signal‐time curves (Fig. 3c) follow a similar pattern to simulated signal‐time curves (Fig. 3b) confirming that the dimension of the signal‐time curves and gas‐flow rates used in simulations are in agreement with the phantom imaging. Experimental signal‐time curves captured using the *N*_int_ = 2 spiral approach in (Fig. 3d) are not as good as the *N*_int_ = 1 spiral because of the time delay (*t*_*d*_) of 500 ms between the I and II interleaves. The temporal resolution of the *N*_int_ = 2 spiral approach can be improved by reconstructing interleaves I and II independently as shown in Figure 3e, but at a cost of decreased spatial resolution. Figure 3f shows the line profile of the helix tube (Fig. 1a) for *N*_int_ = 1, 2, 4, and 8 enabling comparison of their spatial resolutions. The average FWHM of all peaks from H1 to H5 are 10.0 ± 2.5 mm, 6.8 ± 0.6 mm, 6.1 ± 0.6 mm, and 5.5 ± 0.5 mm for *N*_int_ = 1, 2, 4, and 8, respectively. The results indicate that the *N*_int_ = 2 spiral offers comparable spatial resolution to *N*_int_ = 4 and *N*_int_ = 8 spirals, but improved temporal resolution.

**Figure 4:**
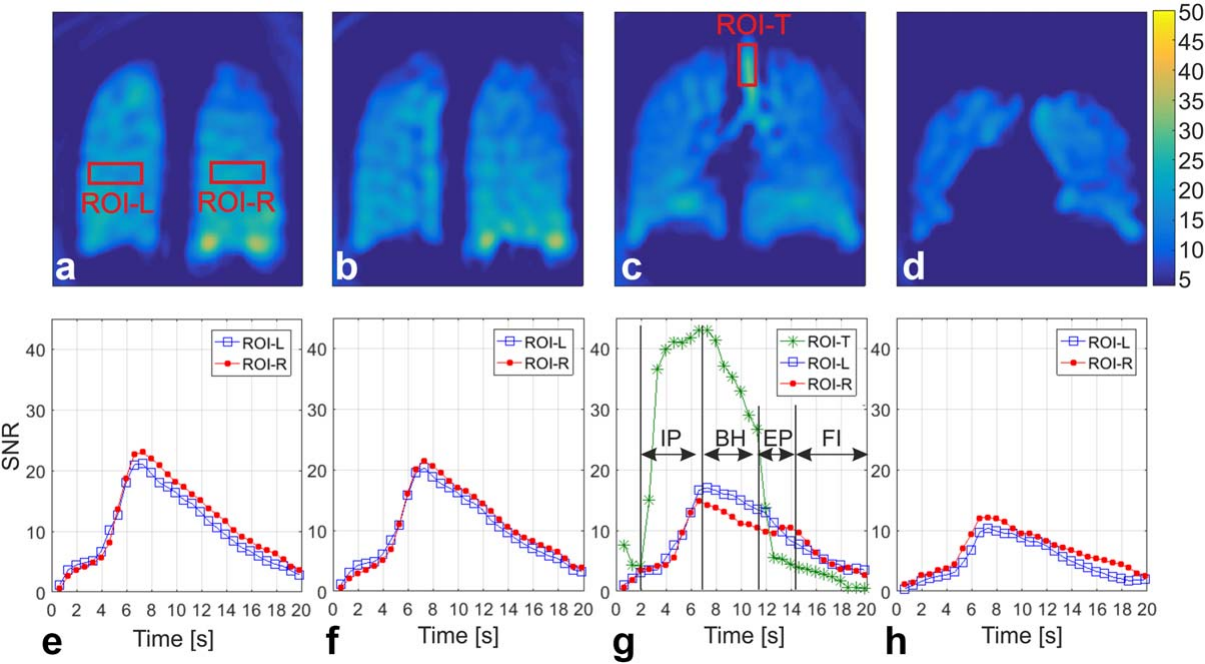
Representative coronal DXeV images of the first healthy volunteer (43‐year‐old male) obtained with the *N*_int_ = 2 spiral approach (**a**) slice 2, (**b**) slice 3, (**c**) average of slices 5, 6, and 7 for a full coverage of trachea, and (**d**) slice 9. Corresponding signal time curves of ROIs from the left lung (ROI‐L) and right lung (ROI‐R) as shown in (**a**) are also plotted from (**e**) to (**h**). ROI‐T stands for signal time curves of an ROI from trachea as shown in (**c**). IP, inhalation period; BH, breath‐hold; EP, exhalation period; FI, flushing interval in (**g**).

Figure 4 shows an *N*_int_ = 2 spiral DXeV images successfully acquired from the first healthy volunteer. DXeV images enabled signal‐time curves to be captured with a very high temporal resolution (i.e., 13 coronal slices within 624 ms). Representative coronal DXeV images from slices 2 and 3 are shown in Figures 4a,b that were averaged over eight time frames during the breath‐hold interval corresponding to eight time points in the signal‐time curves as shown in Figure 4g. Corresponding signal‐time curves from ROI‐Left and ROI‐Right are shown in Figures 4e,f. Slice 2 and Slice 3 in Figures 4a,b represent two consecutive coronal slices located in the posterior lung regions. The image intensity distribution is homogeneous in the posterior regions because these are located some distance from the trachea and large bronchioles, resulting in similar signal‐time curves trends when ROI‐L (Fig. 4a) and ROI‐R (Fig. 4a) are compared between Slice 2 and Slice 3. The average of the three central lung slices (Slices 5, 6, and 7) over eight time frames during the breath‐hold interval are shown in (Fig. 4c). The high signal from the trachea in Figure 4c and the corresponding signal‐time curves at Slice 6 from the ROI trachea is shown in Figure 4g. HP ^129^Xe gas arrives in the trachea at ∼2.0 s and saturates at 6.5 s, corresponding to the signal elevation during the inhalation‐period. HP ^129^Xe gas initially saturates within the trachea following a pattern similar to the signal‐time curves of the gas‐flow phantom because the speed of gas and gas‐flow geometry are comparable to that within the helix tube of the phantom. Following saturation within the trachea, the concentration of HP ^129^Xe gradually increases in the left and right lungs reaching a maximum at 6.5 s. During the breath‐hold, HP ^129^Xe signal loss occurs as a function of the number of RF pulses applied from 6.5 s to 11 s. The sudden decrease of the trachea signal corresponds to the exhalation of HP ^129^Xe gas during the exhalation period. Although most of the HP ^129^Xe gas is exhaled during the exhalation period, some residual ^129^Xe gas in the lungs may be detected during the flush‐interval as a result of the high temporal resolution and use of high flip angle. Signal‐time curves from posterior (Slice 2) to anterior (Slice 9) lung regions are also shown in Figures 4d–h. The flip angles were determined to be 10.2°, 8.0°, and 8.5° for ROI‐L Slices 2, 6, and 9, respectively, with an uncertainty of approximately ±0.5° for the first healthy volunteer.

**Figure 5:**
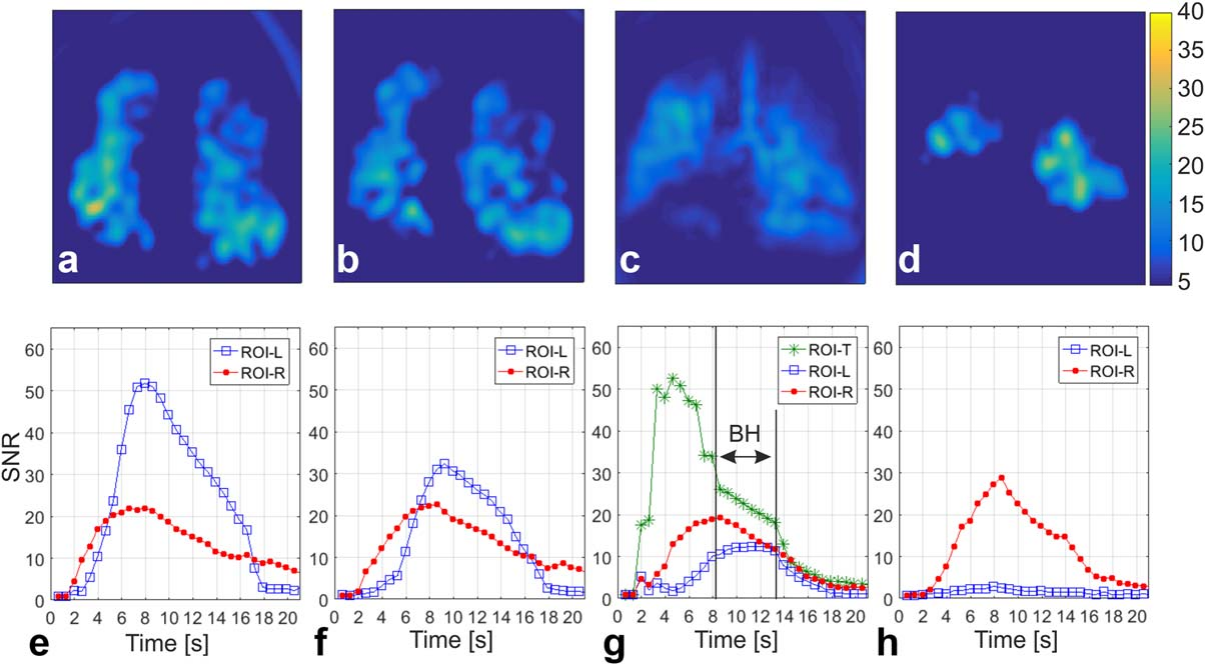
Representative coronal DXeV images of the first COPD patient obtained with the *N*_int_ = 2 spiral approach (**a**) slice 2, (**b**) slice 3, (**c**) average of slices 5, 6, and 7 for a full coverage of trachea, and (**d**) slice 9. Corresponding signal time curves of ROIs from the left lung (ROI‐L) and right lung (ROI‐R) as shown in (**a**) are also plotted from (**e**) to (**h**). ROI‐T stands for signal time curves of an ROI from trachea as shown in (**c**).

DXeV images from a COPD subject and corresponding signal‐time curves are also shown in Figures 5a–d,e–h, respectively. Similar to the healthy subject coronal images, the COPD subject coronal images were also averaged over eight time frames during the breath‐hold interval as eight data points shown in (Fig. 5g). As expected, image intensity in Figures 5a–d is not as homogenous as for a healthy subject (Fig. 4a–d). The signal‐time curves in (Fig. 5e–h) are less repeatable and smooth compared to the signal‐time curves from healthy volunteers in Figures 4e–h.

To demonstrate proof‐of‐concept that DXeV imaging is sensitive to signal‐time curves, signal‐time curves were obtained from three healthy and three COPD subjects and compared in Table 3 The Spearman's correlation coefficient between ROI‐L‐Slice 2 and ROI‐L‐Slice 3, ROI‐L‐Slice 5, ROI‐L‐Slice 9, ROI‐R‐Slice 2, ROI‐R‐Slice 3, ROI‐R‐Slice 5, and ROI‐R‐Slice 9 was determined for each subject by controlling for the ROI‐T (Table 3). As expected, there is not any statistical difference (*P* > 0.05) in rho values across the healthy subjects. A strong correlation was observed between the consecutive image slices (ROI‐L‐Slice 2 and ROI‐L‐Slice 3) for all three healthy subjects. The correlation was weakest when posterior regions of left lung (ROI‐L‐Slice 2) were compared to anterior regions of the right lung (ROI‐R‐Slice9) in all healthy volunteers. The paired samples *t* test failed to reveal a statistical difference between the mean rho values from healthy subject and each COPD subjects (*P* < 0.05). As expected, the correlations were much weaker in COPD subjects compared to the healthy subjects as shown in Table 3.

**Table 3:** Summary of the Spearman's Rho Values in Three Healthy and Three COPD Subjects that Measures the Association between the Signal Time Curves of Slice 2 ROI‐Left and Other ROIs while Controlling the Signal Time Curves of Trachea

	Subject No.	Slice 3 ROI‐Left	Slice 5 ROI‐Left	Slice 9 ROI‐Left	Slice 2 ROI‐Right	Slice 3 ROI‐Right	Slice 5 ROI‐Right	Slice 9 ROI‐Right
Healthy subjects	1	0.990	0.973	0.937	0.968	0.975	0.910	0.900
	2	0.991	0.951	0.951	0.987	0.986	0.955	0.946
	3	0.980	0.973	0.941	0.990	0.957	0.961	0.940
	M ± SD	0.99 ± 0.01	0.97 ± 0.01	0.94 ± 0.01	0.98 ± 0.01	0.97 ± 0.02	0.94 ± 0.03	0.93 ± 0.02
COPD subjects	1	0.901	0.894	0.685	0.905	0.848	0.953	0.920
	2	0.670	0.375	0.658	0.707	0.706	0.643	0.631
	3	0.888	0.856	0.751	0.985	0.960	0.823	0.843
	M ± SD	0.82 ± 0.13	0.71 ± 0.29	0.70 ± 0.05	0.86 ± 0.14	0.84 ± 0.13	0.81 ± 0.16	0.80 ± 0.15

M, mean; SD, standard deviations; ROI, region of interest.

## DISCUSSION

In this study, the temporal and spatial resolutions of DXeV imaging in a gas‐flow phantom were investigated using the interleaved spiral k‐space sampling approach correlating to the finite element modelling of gas flow. *N*_int_ = 2 was found to be the most efficient for capturing the signal‐time curves, and has been tested for acquisition of DXeV images and signal‐time curves in three healthy volunteers and three COPD subjects. The rapid DXeV imaging approach with an *N*_int_ = 2 enables full lung coverage (13 coronal slices) within a very short period (temporal resolution of 624 ms). Because a smaller number of views (i.e., two RF pulses) were used to obtain a single slice image, the *N*_int_ = 2 approach also enabled the use of a high flip angle (∼10°), resulting in sufficient SNR during the inhalation and exhalation periods when the concentration of ^129^Xe is low. Therefore, both high temporal resolution and flip angle have allowed measurement of signal‐time curves during the entire breathing cycle.

The tradeoff between DXeV imaging spatial and temporal resolution, including the dependency of interleave numbers on capturing signal‐time curves, was analyzed using the gas‐flow phantom. The simulated ***C_Xe_*** curves (Fig. 3a) with time step size of 0.1s correspond to ideal signal‐time curves, which does not include HP gas depolarization and relaxation terms in Eq. (1). If the TR was defined to be 0.5 s, comparable to the achievable temporal resolution in multi‐slice clinical imaging, and if the depolarization and relaxations were included, the simulated signal‐time curves (Fig. 3b) followed a similar pattern to those of measured signal‐time curves (Fig. 3c). Because the tube diameter and flow‐rate were the same between each ROI, the amplitude of signal‐time curves from H1 to H5 showed a decreasing trend in relation to the number of RF pulses applied. Therefore, accurately capturing the actual signal‐time curves simulated with a temporal resolution of 0.1s depends on the number of RF pulses (i.e., sampling approach) and the MR timing parameters (TR, *t*_d_, T_read_) because of the gas inflow effect. $T_{2}^{*}$ is assumed to be constant for modelling the gas‐flow phantom in Eq. (1), because the xenon gas is saturated in <1 s at ROIs as shown in Figure 3a. However, saturation of xenon gas at ROIs for those taking longer times may result in significant variations of $T_{2}^{*}$. Specifically, these variations of $T_{2}^{*}$ would be expected to take place during the inhalation period of ∼3 s and may need to be considered when modelling signal‐time curves in human lungs.

There are, however, spatial resolution and susceptibility limitations with spiral interleave k‐space sampling that play an important role in capturing the signal‐time curves. Although the *N*_int_ = 1 spiral has a 2‐fold higher temporal resolution, the *N*_int_ = 2 spiral approach achieves better spatial resolution as shown in the gas‐flow phantom images. In addition, the *N*_int_ = 2 method confers ∼30% reduction in susceptibility and blooming artefacts compared to *N*_int_ = 1 spiral. The blooming artefact similar to that reported by Saam et al. 20 may have caused the discrepancy between the simulated and experimental signal‐time curves H1 in Figures 3b,c. H1 to appear larger in the image (Supporting Fig. S1a; t = 4 s) but to have lower signal intensity (H1 on Fig. 3c) and broader FWHM (H1 on Fig. 3f) compared to other ROIs. The source of the susceptibility artefacts was not investigated in this study; B_o_ distortions have been reported to cause the blooming artefact 47.

Although the *N*_int_ = 4 and *N*_int_ = 8 sampling strategies offered better spatial resolution than the *N*_int_ = 2 approach by ∼20% and 30%, respectively, they were not capable of capturing signal‐time curves because of their inherent low temporal resolution. In principle, the *N*_int_ = 1 spiral was the best approach for capturing the signal‐time curves and correlated most strongly with the simulated signal‐time curves. However, application of *N*_int_ = 1 spiral under clinical conditions proved challenging and impractical because of the associated susceptibility artefacts and limited spatial resolution. I and II interleaves may be reconstructed as separate images to further improve the temporal resolution of the *N*_int_ = 2 spiral approach by a factor of two, while maintaining the spatial resolution for the reconstruction of I and II interleaves together 40. The temporal resolution of signal‐time curves obtained from I and II interleaves following separate reconstruction were visually comparable to the *N*_int_ = 1 spiral. However, signal‐time curves were limited by the additional noise caused by image reconstruction artefacts because of the reduced number of k‐space points from each of the interleaves.

The relatively lower gyromagnetic ratio and consequently longer $T_{2}^{*}$ of ^129^Xe gas in vivo allows the number of interleaves to be reduced without encountering significant susceptibility artefacts because of B_o_ distortions that were previously reported to be a considerable limiting factor for dynamic HP ^3^He‐MR ventilation imaging 16. Spatial susceptibility artefacts were shown to reduce as the number of interleaves was increased to 24, while maintaining a spatial resolution that is comparable to conventional Cartesian k‐space sampling. However, the temporal resolution drops significantly for multi‐slice imaging of whole lungs using a multi‐interleaved spiral. For example, for a TR_min_ = 23 ms with a *N*_int_ = 8 spiral covering 13 coronal slices, the total scan time required per volume would be 2.4 s (Table 1). This would not provide adequate time to capture a sufficient number of dynamic images during the inhalation and exhalation period, each lasting ∼3 s. Although shorter values of T_read_ per interleaf would enable an increased temporal resolution, this would result in both reduced spatial resolution and SNR. This effect has been previously explained using the concept of point spread function (PSF) and amplitude loss as a function of T_read_/ $T_{2}^{*}$ 18. As expected, a high number of interleaves (*N* = 24) requires the use of a low flip angle (α < 5°) for dynamic imaging (for a set of 32 volume images) because the total number of RF pulses is expected to be very large (*N* = 24 × 32). The use of a low flip angle (α < 5°) would also particularly limit SNR for capturing the signal‐time curves where the HP ^129^Xe concentration within the lungs during the inhalation and exhalation‐period was very low.

Signal‐time curves have also been influenced by the subjects' breathing, potential regional differences in oxygen partial pressure and xenon concentration, residence time of the gas, and RF pulse history 15. The effects of those changes on signal‐time curves are to be investigated and measured in a gas flow phantom in corroboration to the 3D gas flow numerical model in future. In this study, the feasibility of a HP ^129^Xe rapid multi‐slice imaging technique was reported using a spiral k‐space sampling approach ignoring the effects of T_1_ and T_2_ variations, the variations between oxgen partial pressure and xenon concentration, residence time of the gas, and RF pulse history.

## CONCLUSIONS

Phantom scans in conjunction with gas‐flow modelling were carried out to investigate the optimum number of spiral k‐space interleaves to achieve sufficient temporal and spatial resolutions. The two‐interleaved spiral (*N*_int_ = 2) was found to be the most efficient strategy for capturing the DXeV images and signal‐time curves. The successful acquisition of DXeV images using *N*_int_ = 2 spiral k‐space sampling approaches has been demonstrated in three healthy and three COPD subjects. The *N*_int_ = 2 spiral approach provided high temporal resolution (624 ms for 13 slices) and used a high flip angle (∼10°), to capture signal‐time curves from selected ROIs. The signal‐time curves were repeatable, smooth, and statistically not different between the heathy volunteer subjects (*P* > > 0.05). The signal‐time curves from COPD subjects associated with obstructive flow were found to be statistically different (*P* < 0.05) from the normal signal‐time curves in healthy subjects.

## Supporting information


**Fig. S1**. (**a**) DXeV images of gas‐flow phantom were obtained with TR = 500 ms using N_int_ = 1 and (**b**) N_in t_ = 2. Susceptibility artefacts are shown with arrow in (**a**) at time points of *t* = 5 s and *t* = 5.5 s. H1 and H5 are shown with arrows in (**a**) at time point of *t* = 4 s.
