# Supplementary material for: Fast dynamic ventilation MRI of hyperpolarized 129Xe using spiral imaging
Source: Magn Reson Med. 2017 Sep 16;79(5):2597–606. doi: 10.1002/mrm.26912 (PMC5836876; doi:10.1002/mrm.26912)
Supplement: Supplementary file 1 — Fig. S1. (a) DXeV images of gas‐flow phantom were obtained with TR = 500 ms using Nint = 1 and (b) Nin t = 2. Susceptibility artefacts are shown with arrow in (a) at time points of t = 5 s and t = 5.5 s. H1 and H5 are shown with arrows in (a) at time point of t = 4 s. [file MRM-79-2597-s001.doc]

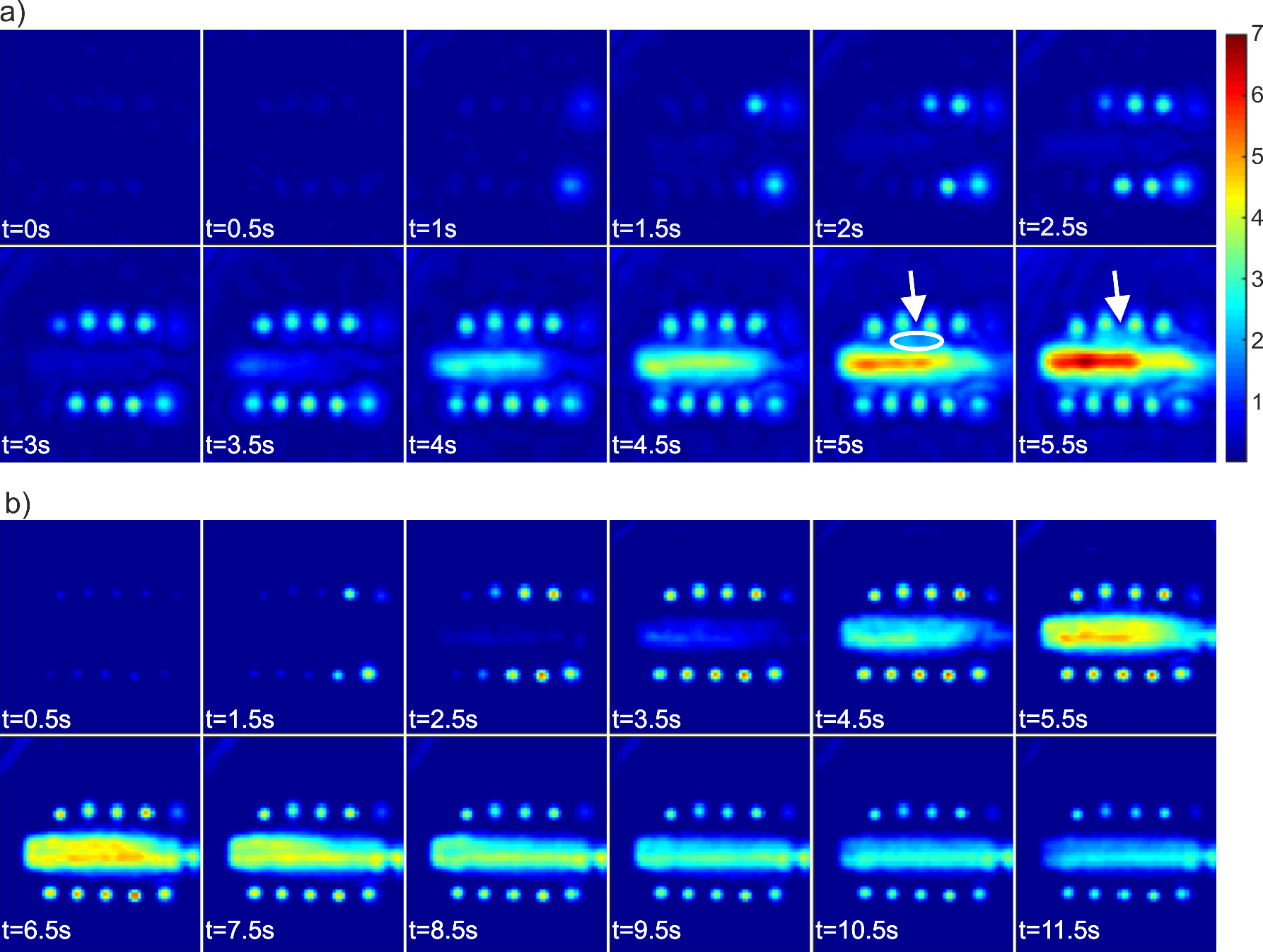


**Supporting Figure S1:** (a) DXeV images of gas-flow phantom were obtained with TR = 500 ms using N_int_= 1 and (b) N_int_= 2. Susceptibility artefacts are shown with arrow in (a) at time points of t = 5 s and t = 5.5 s. H1 and H5 are shown with arrows in (a) at time point of t = 4 s.

**Text for the Supporting Figure S1:**

DXeV images of the gas-flow phantom for *TR*=500ms using *N_int_*=1 and *N_int_*=2 spirals are shown in Supporting Figure S1 (a-b). Variations of DXeV image intensity between images in (a) correspond to the HP ^129^Xe gas flow within the helix tube and syringe body following simultaneous commencement of the power injector and MRI scanner. Conceptually for corresponding time-points, the simulated
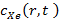
 map in Figure 1(b) is comparable to the phantom image in Supporting Figure S1(a) at *t*=3.5s excluding the effects of the flip angle, noise, relaxation times, sampling, and MRI artefacts. ^129^Xe gas does not appear in the first (*t*=0s) and second (*t*=0.5s) images during the transit time within the extension tube. Flow of HP ^129^Xe gas from H1 to H5 is captured from the fourth image (*t*=1.5s) to the ninth image (*t*=4.0s) in Supporting Figure S1 (a). The image intensity in the central core of the syringe body is apparent on the 8^th^ through 12^th^ images which are similar to the simulated
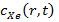
 map in Figure 1(b). Susceptibility artefacts are apparent at the 5s time-point near the helix tube and syringe body interface. Susceptibility artefacts are made more apparent by the high signal within the syringe body. Supporting Figure S1(b) shows DXeV images obtained with *N_int_*=2 and *TR* of 500ms resulting in a delay of 1s between each image.
